# Editorial: The evolution in RNA: 2023

**DOI:** 10.3389/fgene.2024.1457242

**Published:** 2024-07-30

**Authors:** Wei Lan, Yuan Zhou, Rasmani Hazra

**Affiliations:** ^1^ School of Computer Electronical and Information, Guangxi University, Nanning, China; ^2^ Department of Biomedical Informatics, School of Basic Medical Sciences, Peking University, Beijing, China; ^3^ Department of Biology and Environmental Science, University of New Haven, West Haven, CT, United States

**Keywords:** RNA, disease, RNA modification, function enrichment analysis, data integration

With the development of Next-Generation Sequencing (NGS) technologies, more and more RNAs have been identified. They can be classified into coding RNA and non-coding RNA. In the past long time, non-coding RNA has been characterized as genomic “junk.” However, many studies have proved that it also plays key roles in many important biological processes. RNAs have a close relationship with the regulation, expression, and evolution of genetic information. To provide a platform for exhibiting the latest technology regarding RNA, we organized a Research Topic on “The Evolution in RNA: 2023.” This Research Topic presents four articles. We expect that these articles will promote more advanced studies in RNA research.

RNA modification is emerging as an important new layer of post-transcriptional regulation of the transcriptome. More than a hundred types of RNA modifications have been discovered, but only a few of them allow transcriptome-wide profiling. Dihydrouridine (D) modification, a conserved oxidoreduction RNA modification type in tRNAs and a few mRNAs, is one of the latest RNA modifications that can be profiled at the transcriptome level. In this Research Topic, Ren et al. have comprehensively encoded the sequence features of D modification and established a multi-species prediction method for D modification. With this predictor, it will be possible to more accurately predict D modification based on RNA sequences alone.

With the development of high-throughput techniques, the definition of non-coding RNAs has evolved. That is, more and more peptides and small proteins in the proteome are produced by non-coding RNAs. Among these non-coding RNAs, circular RNAs (circRNAs) are emerging as an interesting category with relatively extensive coding potential. In this Research Topic, Shi et al. have systematically reviewed the recent progress of protein-coding circRNAs since 2022. This review outlines the mechanisms, research progress, and prediction models of circRNA-coding proteins. They found that although oncogenesis and tumor suppression are major focuses, circRNAs also affect cardiovascular, muscular, and nervous system diseases. Some helpful perspectives for future research were also provided, including but not limited to the need for more in-depth validation through rescue assays and the urgent need for investigation beyond cancers.

MicroRNAs (miRNAs) are a class of non-coding RNA molecules that play critical roles in a variety of biological processes. In this Research Topic, Li et al. proposed a new miRNA set enrichment analysis method (MHIF-MSEA) based on multi-source heterogeneous information fusion. They constructed three miRNA similarity networks (miRSN-DA, miRSN-GOA, and miRSN-PPI) based on miRNA-disease association, gene ontology (GO) annotation of target genes, and protein-protein interaction of target genes, respectively. In addition, the authors integrated these miRNA similarity networks into a single similarity network by using an averaging method. The random walk with restart algorithm was used to expand the original miRNA list based on the fused network Finally, the researchers performedenrichment analysis on the expanded list. This work will be useful for miRNA functional enrichment analysis.

Glioblastoma is a type of aggressive brain tumor with a very poor survival prognosis due to its tumorigenic stem cells (GSCs). Long non-coding RNAs (lncRNAs) have attracted considerable interest due to their critical roles in normal physiological processes and cancer development. Extensive research has highlighted their significant influence in regulating the properties of GSCs. In this Research Topic, Hazra et al. systemically reviewed the role of long non-coding RNA in glioblastoma. First, they delved into some notable examples of lncRNAs related to glioblastoma stem cells, and elucidated their functional roles in driving glioblastoma progression. Then, they further explored the use of a 3D *in vitro* model to investigate GSC biology. In addition, they elucidated four primary methods for targeting lncRNAs as potential therapeutics in the management of glioblastoma.

